# Clinical outcomes following pre-, pro- and synbiotic supplementation after caesarean birth or antibiotic exposure in the first week of life in term born infants: A systematic review of the literature

**DOI:** 10.3389/fped.2022.974608

**Published:** 2022-10-10

**Authors:** Kim Kamphorst, Nora C. Carpay, Tim G. J. de Meij, Joost G. Daams, Ruurd M. van Elburg, Arine M. Vlieger

**Affiliations:** ^1^ Department of Pediatrics, Amsterdam UMC, Location University of Amsterdam, Amsterdam Gastroenterology and Metabolism Research Institute, Amsterdam, Netherlands; ^2^ Department of Pediatrics, St. Antonius Hospital, Nieuwegein, Netherlands; ^3^ Department of Pediatric Gastroenterology, Amsterdam UMC, Location Vrije Universiteit Amsterdam, Amsterdam, Netherlands; ^4^ Medical Library, Amsterdam UMC, Location University of Amsterdam, Amsterdam, Netherlands

**Keywords:** neonate, allergy, supplementation, probiotic, prebiotic, synbiotic

## Abstract

**Background:**

Caesarean section and early exposure to antibiotics disrupt the developing gastrointestinal microbiome, which is associated with long-term health effects.

**Objective:**

The aim of this systematic review was to summarise the impact of prebiotics, probiotics, or synbiotics supplementation on clinical health outcomes of term infants born by caesarean section or exposed to antibiotics in the first week of life.

**Design:**

A systematic search was performed in Medline and Embase from inception to August 2021. Title and abstract screening (*n* = 11,248), full text screening (*n* = 48), and quality assessment were performed independently by two researchers.

**Results:**

Six RCTs studying caesarean born infants were included, group sizes varied between 32–193 with in total 752 children. No studies regarding supplementation after neonatal antibiotic exposure were found. Three studies administered a probiotic, one a prebiotic, one a synbiotic, and one study investigated a prebiotic and synbiotic. Several significant effects were reported at follow-up varying between 10 days and 13 years: a decrease in atopic diseases (*n* = 2 studies), higher immune response to tetanus and polio vaccinations (*n* = 2), lower response to influenza vaccination (*n* = 1), fewer infectious diseases (*n* = 2), and less infantile colic (*n* = 1), although results were inconsistent.

**Conclusions:**

Supplementation of caesarean-born infants with prebiotics, probiotics, or synbiotics resulted in significant improvements in some health outcomes as well as vaccination responses. Due to the variety of studied products and the paucity of studies, no recommendations can be given yet on the routine application of prebiotics, probiotics, or synbiotics to improve health outcomes after caesarean section or neonatal antibiotic exposure.

## Introduction

Early life is an important period as the infant's immune system is still developing (1). The development of the immune system is influenced by the gut microbiome (1), which develops rapidly after birth (2). Disruption of the developing gut microbiome (dysbiosis) due to environmental factors have been associated with adverse long-term health effects (3, 4).

Caesarean section (CS) is one of the main causes of aberrant microbiome development because it affects the diversity and colonization pattern of the gut microbiome (5–7). Due to reduced vertical mother-infant transmission of beneficial gut bacteria, the infant is predominantly colonized with bacteria from the skin, mouth and hospital environment (8–14). This is associated with an altered immune development, a higher risk of childhood obesity, atopy, allergy, asthma, and type 1 diabetes mellitus (10, 15, 16).

Another important cause of early-life dysbiosis is antibiotic exposure (17–19). Antibiotics are the most frequently prescribed drugs for neonates in their first week of life (20, 21), but their effects on later health outcomes have not yet been fully elucidated. So far, a few observational studies have shown that infants exposed to antibiotics in their first week of life had an altered gut microbiota (22–25) and it was associated with an increased risk of wheezing (26–28), infantile colic (26), gastrointestinal disorders (29) impaired growth (22, 30), allergies (31), allergic rhinitis (27), functional abdominal pain (32) and asthma (33, 34).

Potential interventions to reduce some of these long-term effects of early life dysbiosis include supplementation with prebiotics, probiotics, or synbiotics. Prebiotics are nutrients that promote growth and activity of beneficial bacteria that already exist in the gut (35), probiotics are live microorganisms such as Bifidobacteria and Lactobacilli (13), and synbiotics are a combination of pro- and prebiotics (36). The aim of this systematic review was to summarise the impact of prebiotics, probiotics, or synbiotics supplementation on clinical health outcomes of term infants born by caesarean section or exposed to antibiotics in the first week of life.

## Methods

### Literature search

This systematic review was conducted according to the guidelines of the Preferred Reporting Items for Systematic Reviews and Meta-Analyses (37). OVID Medline and Embase were systematically searched from inception to 3 August 2021. A multi stranded search approach comprised the following concept combinations:

([c section] OR ([antibiotic treatment] AND [first week of life] OR [first week antibiotics])) AND

- [pre- pro- synbiotics]
OR
- [dietary supplements] AND [microbiome]
OR
- [dietary supplements brands]

To reduce recall bias and enhance search results precision VOS-viewer was used to identify terms for NOTing out irrelevant records from databases searched (38, 39). No other filters or limits were used (Supplementary Appendix S1).

#### Inclusion criteria

(1) study participants were term-born infants (born between 37 and 42 weeks of gestation) and born *via* caesarean section or exposed to antibiotics in the first week of life; (2) exposure to pre-, pro- or synbiotic dietary supplements administered within six weeks after birth; (3) clinical outcomes were reported; (4) study design was a randomised controlled trial (RCT).

#### Exclusion criteria

(1) including infants with major congenital malformations; (2) written in a language other than English; (3) animal studies; (4) for the caesarean-analyses: if a study includes both vaginally and caesarean-delivered infants and there were no subgroup analyses for only the caesarean-delivered infants

### Data collection

After the search, all records were imported into Rayyan after deduplication (40). Two researchers (NC and KK) independently performed title and abstract screening, as well as full-text screening. After consensus about the included articles, relevant data were extracted by NC in consultation with the other co-authors. Odds ratios (ORs), 95% confidence intervals (95% CI) and *P*-values were included in the table if these were provided in the original articles. If both “per protocol” and “(modified) intention to treat” analyses were available, only the results from the “(modified) intention to treat” analysis were included.

### Critical appraisal

To assess the risk of bias in the included articles, the Cochrane risk-of-bias tool for randomised controlled trials (RoB 2) (41) was used. The RoB 2 assesses the risk of bias in the studies in five domains (Table 1). The risk of bias was independently assessed by two researchers (NC and KK) and any discrepancies were discussed until a consensus was reached.

**Table 1:** Risk of bias of the included studies.

First author	Domains of the Cochrane risk-of-bias tool for randomised controlled trials (RoB-2)					
	Domain 1	Domain 2	Domain 3	Domain 4	Domain 5	Total
Puccio (48)						
Chua (42)						
Kallio (43)						
Kuitunen (44)						
Peldan (45)						
Baglatzi (46)						
Cooper (49)						
Holscher (47)						

Domain 1: Risk of bias arising from the randomisation process.

Domain 2: Risk of bias due to deviations from the intended interventions (*effect of adhering to intervention*).

Domain 3: Missing outcome data.

Domain 4: Risk of bias in measurement of the outcome.

Domain 5: Risk of bias in selection of the reported result.

Green: low risk of bias, yellow: some risk of bias, red: high risk of bias.

If a study included both vaginally and caesarean-delivered infants and a subgroup analysis on the caesarean-delivered infants was performed, only the methods used for this relevant subgroup analyses were assessed.

### Data analyses

Due to the heterogeneity in the interventions and outcomes evaluated in this systematic review, it is not possible to synthesize data from these studies in a meta-analysis. Therefore, a descriptive synthesis of the data was performed.

## Results

**Figure 1:**
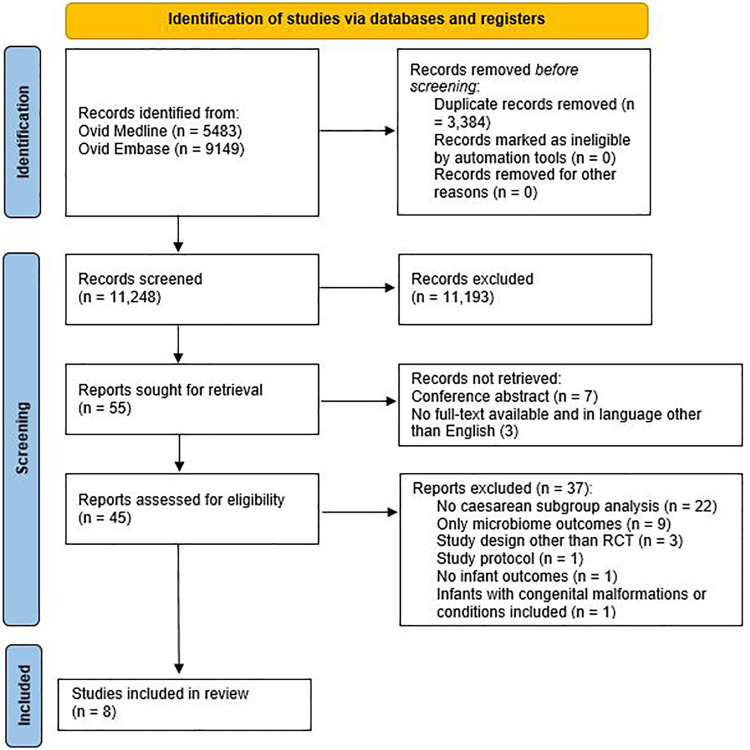
Flowchart showing article selection. Adopted from the PRISMA 2020 flow diagram.

Of the 14,632 records, 11,248 remained after removing duplicates. After title and abstract screening, 55 articles were read in full-text, and eight articles were included for analysis (see Figure 1).

### Study characteristics

Eight articles were included, based on six RCTs (Figure 1), with a total of 752 children. Most studies scored a high risk of bias (Table 1). The characteristics of the included studies are summarised in Table 2. In all studies, supplementation was administrated to infants born by CS; no studies were found after antibiotics in the first week of life. The antibiotic policy for CS was not described in most studies, only Chua et al. (42) stated that infants born via CS were exposed to intrapartum antibiotics prophylaxis. It is likely that in more studies caesarean-born infants were exposed to antibiotics in utero.

**Table 2:** General characteristics of the included studies.

First author	Country	Study period (year published)	# Participants^a^			AB or CS SG?	Intervention	Control	Start of intervention	Duration intervention	Outcomes (relevant subgroup)	Follow-up	Comments
			I	C	T								
Puccio (48)	Italy / Belgium	2012–2015 (2017)	32	32	64	CS SG	Prebiotics: 2 HMOs (2′-fucosyllactose and lacto-N-neotetraose)	Control formula	0–14 D	6 M	Colic, nighttime awakenings, bronchitis, LRTI	1, 2, 3, 4, 6, 12 M	Safety study, CS SG results are only reported if they are significant
Chua (42)	Singapore / Thailand	2011–2013 (2017)	52 + 51	50	153	CS	Prebiotic (scGOS/lcFOS) or synbiotic (scGOS/ICFOS and *Bifidobacterium breve* M-16 V)	Control formula	1–3 D	16 W	**Total faecal Bifidobacteria**, *Bifidobacterium* species abundance, other members of the gut microbiota, pH, sc fatty acids, lactate, atopic dermatitis/eczema	3, 5 D 2, 4, 8, 12, 16, 22 W	Infants born *via* CS were also exposed to intrapartum AB prophylaxis^b^.
Kuitunen (44)	Finland	2000–2003 (2009)	70	76	146	CS SG	Probiotic: *Lactobacillus rhamnosus* LC705, *Bifidobacterium breve* Bb99, *Propionibacterium freudenreichii* spp., *shermanii* JS	Placebo (micro-crystalline cellulose)	36 W gestation + from birth	6 M	**(IgE-mediated) allergic disease**, eczema, food allergy, asthma, allergic rhinitis, IgE sensitisation alone	5 Y	Infants were at risk for atopic diseases (at least one parent with asthma, allergic rhinitis or eczema). The intervention was initiated prenatally (36 W gestation) Kallio et al.: significant differences between sub- and main group: longer BF, older maternal age in CS SG, more use of prebiotics after 13 years in intervention group Peldan et al.: more use of probiotics after the study period in the intervention group
Peldan (45)	Finland	2000–2003 (2017)	69	75	144						**Allergic disease, eczema, allergic rhinitis, asthma**, URTIs, LRTIs, AB use, food allergy	10 Y	
Kallio (43)	Finland	2000–2003 (2019)	53	56	109						Allergic disease (**doctor-diagnosed**, ISAAC), **sensitisation**	13 Y	
Baglatzi (46)	Greece	2009–2011 (2016)	84	80	164	CS	Probiotic: regular dose of *Bifidobacterium lactis*	Low dose of *B. lactis*	Birth	6 M	**Diarrhoea**, immune and gut maturation, microbiota, immune response to vaccines, anthropometry	12 M	No control group that was fed formula without pre/pro/synbiotics
Cooper (49)	South Africa	2008–2013 (2016)	92	101	193	CS SG	Synbiotic: BMOs (containing GOS and MOS such as 3’- and 6’ sialyllactose) + *Bifidobacterium lactis* CNCM-I-3446	Control formula	Birth (≤3 D)	6 M	**Faecal (bifido)bacteria, anthropometrics**, faecal pH, lean mass, fat mass and bone mineral content, digestive tolerance, immune parameters, HIV infection status, frequency of morbidity episodes	1 Y	The included infants all have HIV+ mothers and all mothers and infants received antiretroviral medication. Infants who tested positive for HIV were excluded
Holscher (47)	USA	2007–2008 (2012)	16	16	32	CS SG	Probiotic: *Bifidobacterium animalis* subspecies lactis (Bb12)	Control formula	6 W	12 W	**Faecal sIgA**, anti-rotavirus-specific IgA, faecal anti-poliovirus-specific IgA	12 W	

CS, Caesarean section; SG, subgroup; I, intervention; C, controls; T, total; BF, breastfeeding; FF, formula feeding; HMOs, human milk oligosaccharides; (sc) GOS, (short chain) galactooligosaccharides; (lc) FOS, (long chain) fructooligosaccharides; Spp, several species; BMOs, bovine milk oligosaccharides; MOS, milk oligosaccharides; D, days; M, months; W, weeks; H, hours; Y, year; AB, antibiotic; LRTI, lower respiratory tract infection; URTI, upper respiratory tract infection; ISAAC, International study of asthma and allergies in childhood; HIV, human immunodeficiency virus; sIgA, secretory Immunoglobulin A.

Outcome in **bold** was the primary outcome of the study.

^a^ # participants in a subgroup, if applicable.

^b^ The antibiotic policy for CS was not described in most studies, only Chua et al. (42) stated that infants born *via* CS were exposed to intrapartum antibiotics prophylaxis. It is likely that in more studies caesarean-born infants were exposed to antibiotics *in utero*.

In three articles, based on the same study, the intervention was a probiotic mixture (43–45) [see Table 2)]. In two other studies, the intervention group was also given a probiotic (46, 47) and the interventions of the other three studies were prebiotics (48), synbiotics (49), and either pre- or synbiotics (42). All interventions were started within two weeks after birth, except for one study in which the intervention was started at six weeks of age (47). The intervention was administered for six months in most studies, except for two studies in which the intervention was continued until 12 weeks of age (47) or 16 weeks of age (42). In five RCT's, the intervention group was only compared with the placebo control group and not with the breastfed reference group for the clinical outcomes. Therefore, only the results between the intervention and the control groups are reported.

#### Atopic diseases

Four articles examined the effect of supplementation on atopy. Three articles (43–45) based on the same RCT evaluated the effect of a prenatally started probiotic supplement until six months of age on allergic disease in infants (*n* = 146) at risk for atopic diseases at 5, 10 and 13 years of age. There was no significant difference between the intervention and control group for most outcomes regarding eczema, sensitisation, any allergic disease, and rhinitis until 13 years of follow-up (Table 3). The reported significant results were a decrease in IgE-associated eczema, and a positive (food) skin prick test (SPT) response and/or food-specific IgE >0.7 kU/L at 0–5 years of age in the intervention group (44). At 13 years of age, there was a significant decrease in eczema and any allergic disease experienced in the last 12 months, based on the ISAAC questionnaire (43, 50). The study by Chua et al. (42) examined the effect of a prebiotic and a synbiotic supplementation administrated until 16 weeks of age (*n* = 153). In post-hoc analyses, fewer skin disorders and atopic dermatitis/eczema were found in the synbiotic group, but not in the prebiotic group compared to the control group at 22 weeks.

**Table 3:** Clinical outcomes.

First author	Intervention			Participants^a^			Significant outcomes					Non-significant outcomes	Comments
	Type	Start	Duration	I	C	T	Outcome	Reply/specific outcome	Time point	OR (95% CI)	*P*-value		
**Allergic disease**													
Chua (42)	Prebiotic (scGOS/lcFOS)	1–3 D	16 W	52	50	102	–	–	22 W	–	–	1. All skin disorders at 22 W 2. Atopic dermatitis/eczema at 22W	Post-hoc analysis
	Synbiotic (scGOS/ICFOS and *B. breve* M-16 V)	1–3 D	16 W	51	50	101	Skin disorders	All skin disorders	22 W	–	0.017	–	
								Atopic dermatitis/eczema	22 W	–	0.037		
Kuitunen (44)	Probiotic: *L. rhamnosus* LC705, *B. breve* Bb99, *P. freudenreichii* spp., *shermanii* JS	36 W gestation + from birth	6 M	70	76	146	Allergic disease	**Positive SPT response**	0–5 Y	0.47 (0.23–0.96)	<0.05	1. **Allergic disease: positive SPT response at 0–2 Y, specific IgE >0.7 kU/L at 0–5 Y** 2. Sensitization: positive SPT response and/or specific IgE >0.7 kU/L at 0–2 and 0–5 Y 3. Allergic disease: all eczema at 0–2 and 0–5 Y, IgE-associated eczema at 0–2 Y, IgE-associated asthma and rhinitis at 0–2 and 0–5 Y	–
								Eczema: IgE-associated	0–5 Y	0.43 (0.19–0.95)	<0.05		
							Sensitisation	Positive food SPT response and/or food-specific IgE >0.7 kU/L	0–5 Y	0.33 (0.12–0.85)	<0.05		
Peldan (45)				69	75	144	–	–	–	–	–	1. Any allergic disease ever: ISAAC, doctor-diagnosed, ISAAC at 5–10 Y 2. Eczema ever: ISAAC, doctor-diagnosed, ISAAC at 5–10 Y 3. Allergic rhinitis ever and in the last 5–10Y: ISAAC, doctor-diagnosed 4. Asthma ever: ISAAC, doctor-diagnosed, ISAAC at 5–10 Y 5. Doctor-diagnosed food allergy ever	Allergic rhinitis was significantly decreased in the intervention group in the unadjusted OR
Kallio (43)				53	56	109	Allergic disease (ISAAC, last 12 M)	Allergic disease	13 Y	0.336 (0.154–0.736)	0.006	1. **Allergic disease: any/specific IgE >0.7 kU/L** 2. Sensitization: any/food-specific/inhalant-specific IgE >0.7/>0.35 kU/L 3. Doctor-diagnosed allergy ever: all/IgE-associated eczema, asthma, rhinitis or food allergy 4. ISAAC-diagnosed allergic disease in last 12 M: allergic disease, specific IgE >0.7 kU/L, IgE-associated eczema, asthma and rhinitis	Holm method was used to adjust for multiple comparisons
								Eczema		0.388 (0.162–0.930)	0.031		
**Infectious diseases**													
Puccio (48)	Prebiotics: 2 HMOs (2’-fucosyllactose and lacto-N-neotetraose)	0–14 D	6 M	32	32	64	LRTI	Any	6 M	0.17 (0.02–0.96)	0.043	–	Unclear if other analyses were performed
								Any	12 M	0.21 (0.04–0.83)	0.022		
							Bronchitis	Any	12 M	0.06 (0.00–0.50)	0.003		
Peldan (45)	Synbiotic: *L. rhamnosus* LC705, *B. breve* Bb99, *P. freudenreichii* spp., *shermanii* JS and GOS	36 W gestation + from birth	6 M	69	75	144	No AB	During last 5 years	5–10 Y	3.19 (1.02–9.97	0.046	1. LRTI, ≥1/5 years	ORs are adjusted ORs
							URTI	≥4/year	5–10 Y	0.29 (0.12–0.72)	0.004		
**Gastrointestinal effects**													
Baglatzi (46)	Probiotic: regular dose of *B. lactis* (vs. low dose)	Birth	6 M	84	80	164	–	–	1 Y	–	–	**1. Diarrhoea: prevalence, incidence and number of days of diarrhoea at 1 Y**	high dose considered intervention, low dose considered control
Cooper (49)	Synbiotic: BMOs (containing GOS and MOS such as 3’- and 6’ sialyllactose) + *B. lactis* CNCM-I-3446	Birth (≤3 D)	6 M	92	101	193	Stool consistency	↑ Liquid	10 D, 4 W, 6 W, 3 M, 4 M, 6 M	–	<0.001	1. Frequency of daily stools at 3 D, 10 D, 28 D and 3 M 2. Frequencies of flatulence, spitting up, vomiting, crying, fussing, or colic at 10 D, 28 D, 6 W, 3 M and 4 M	–
								↓ Proportion of days in which hard stool was reported (mean %)		–	0.001		
								↓ Proportion of days with formed stools		–	0.045		
Puccio (48)	Prebiotics: 2 HMOs (2’-fucosyllactose and lacto-N-neotetraose)	0–14 D	6 M	32	32	64	Colic	↓ Colic reported	4 M	–	0.035	–	Unclear if other analyses were performed
**Anthropometrics**													
Baglatzi (46)	Probiotic: regular dose of *B. lactis* (vs. low dose)	Birth	6 M	84	80	164	–	–	1 M, 4 M, 12 M	–	–	1. Weight-for-age, length-for-age, BMI-for-age and head-circumference-for-age at 1 M, 4 M and 12 M	high dose considered intervention, low dose considered control
Cooper (49)	Synbiotic: BMOs (containing GOS and MOS such as 3’- and 6’ sialyllactose) + *B. lactis* CNCM-I-3446	Birth (≤3 D)	6 M	92	101	193	**Daily weight gain**	**Between 10 D and 4 M (mean)**	4 M	–	0.010 (Non- inferiority *P*-value)	1. Weight-for-age, length-for-age, BMI-for-age and head-circumference-for-age at 10 D, 4 W, 6 W, 3 M, 4 M and 6 M 2. Fat mass and percentage mean fat mass at 4 M and 12 M	Body weight was adjusted for baseline value and sex Non-inferiority analysis
Behaviour													
Puccio (48)	Prebiotics: 2 HMOs (2’-fucosyllactose and lacto-N-neotetraose)	0–14 D	6 M	32	32	64	Nighttime awakenings	↓	2 M	–	0.036	–	Unclear if other analyses were performed
**Vaccination response**													
Baglatzi (46)	Probiotic: regular dose of *B. lactis* (vs. low dose)	Birth	6 M	84	80	164	Immune responses to vaccinations	↑ Response to Tetanus [lU/ml, median (n; 25th–75th percentile)]	12 M	–	0.0411	1. Immune responses to vaccination at 7 M and 1 Y: diphteria, B. pertussis, tetanus, polio, infants who reached anti-HiB protective antibody over the 12 M	high dose considered intervention, low dose considered control
								↓ Response to H. influenza B [µg/ml, median (n; 25th–75th percentile)]		–	0.0186		
Cooper (49)	Synbiotic: BMOs (containing GOS and MOS such as 3’- and 6’ sialyllactose) + *B. lactis* CNCM-I-3446	Birth (≤3 D)	6 M	92	101	193	–	–	6 W, 4 M, 12 M	–		1. Immune measurements: positive anti-hepatitis B IgG antibody response	–
Holscher (47)	Probiotic: *B. animalis* subspecies lactis (Bb12)	6 W	12 W	16	16	32	Immune responses to vaccinations	↑ anti-polio-specific IgA (U/g) after vaccination	12 W compared to 8 W	–	0.026	1. Change in anti-rotavirus-specific IgA after vaccination between 8 W and 12 W	–

I, intervention; C, controls; T, total; CS, Caesarean section; SG, subgroup; OR, odds ratio; HMOs, human milk oligosaccharides; (sc) GOS, (short chain) galactooligosaccharides; (lc) FOS, (long chain) fructooligosaccharides; BMOs, bovine milk oligosaccharides; MOS, milk oligosaccharides; SPT, skin prick test; ISAAC, International study of asthma and allergies in childhood; D, days; M, months; W, weeks; H, hours; Y, year; AB, antibiotic; LRTI, lower respiratory tract infection; URTI, upper respiratory tract infection.

Outcome **in bold** was the primary outcome of the study.

^a^ # participants in a subgroup, if applicable.

#### Infectious diseases

Two studies (45, 48) examined the effects of prebiotic (48) or synbiotic (45) supplementation in the first six months of life on infectious diseases. Puccio et al. found that infants (*n* = 64) in the prebiotic intervention group had a lower risk of lower respiratory infection at 6 months OR 0.17 (95% CI, 0.02–0.96), or 12 months OR 0.21 (95% CI, 0.04–0.83) or bronchitis at 12 months OR 0.06 (95% CI, 0.00–0.50) than those in the control group (48). Peldan et al. found after 5–10 years follow-up (*n* = 144) that the probiotic intervention was associated with a reduced risk of receiving antibiotics over the past five years OR 3.19 (95% CI, 1.02–9.97) and a lower risk of having four or more upper respiratory infections in one year 0.29 (95% CI, 0.12–0.72) (45).

#### Gastrointestinal effects

Three articles assessed the effect of a prebiotic (48), probiotic (46), and a synbiotic (49) supplementation in the first six months of life on diarrhea (46), stool pattern (49) and colic (48) in the first year of life. Cooper et al. found up to 6 months of age, more liquid stools and fewer formed and hard stools were reported in the probiotic group compared to the control group (*n* = 193) (49). Baglatzi et al. (*n* = 164) found no differences in diarrhoea during the first year (46). Puccio et al. (*n* = 64) found a significantly lower incidence of parent-reported infantile colic at four months of age in the intervention group, which was collected in a diary with the options “never,” “sometimes,” and “often”.

#### Anthropometrics

Two studies (46, 49) examined the effect of a probiotic (*n* = 164) (46) and synbiotic (49) supplement (*n* = 193) on anthropometric measurements during the first year of life. Both studies found no differences in anthropometric measurements including weight-for-age, length-for-age, BMI-for-age, head-circumference-for-age and fat mass between intervention and control group infants (46, 49).

#### Behaviour

Puccio et al. (48) found significantly fewer parent-reported night time awakenings at 2 months in the prebiotic group compared to the placebo group (*n* = 64) (48). Parents reported these awakenings as “never,” “sometimes,” and “often”. The difference did not persist after two months of age.

#### Immune response

Three studies (46, 47, 49) investigated the effect of a probiotic (46, 47) or a synbiotic (49) supplement on the infants' immune system. Holscher et al. (47) found after probiotic supplementation between six and twelve weeks of age a significantly higher increase in anti-polio-specific IgA after vaccination at 12 weeks compared to 8 weeks (*n* = 32). Baglatzi et al. (46) found after six months of probiotic supplementation a significantly higher immune response to tetanus vaccinations (*n* = 164), but a lower immune response to H. influenza B vaccinations at 12 months for the regular dose group compared to the low dose group. In contrast to Holscher at al. (47), no significant differences in immune response to polio vaccinations was found (46). Cooper et al. (49) found no significant differences after synbiotic supplementation (*n* = 193).

#### Safety

All the included studied reported safety in terms of growth and gastrointestinal tolerance and none noted significant differences in these parameters or in the number of adverse events between the intervention and the control group.

## Discussion

This systematic review on the clinical effects of pre-, pro- or synbiotic supplementation after CS or antibiotic exposure in the first week of life shows several significant differences in clinical outcomes. The reported effects consisted of a decrease in atopic diseases, fewer infectious diseases, and difference in immune response to vaccinations. The results with regard to immune response to vaccinations were however inconsistent and only shown in CS born children. No studies were found regarding the effects of pre-, pro- or synbiotics supplementation on clinical outcomes after neonatal antibiotic treatment.

Only one RCT was included in this review in which allergy was the primary outcome (44). It showed some promising results of probiotics for CS born children in a post-hoc analysis, but not for vaginally born children (43, 44). Both this RCT and the study of Chua et al. (42) showed that caesarean-born children in the intervention group had less eczema. The mechanisms behind the prevention of eczema following probiotics stem from the hygiene hypothesis, where early exposure to gut microbes directs the immune system away from a Th-2 skew (51) or upregulates Tk1-cytokine production (52). The protective effects of prebiotics may be by promoting bacterial growth of by immunomodulatory effects (52). Eczema in early life is an important risk factor itself for later allergy development (53), probably due to epicutaneous sensitization. We hypothesize that if pre-, pro- or synbiotic administration reduce the incidence of eczema, these children may have less atopic diseases later in life. Adequately powered studies on the effect of probiotic supplementation in children born following CS are needed to confirm this hypothesis.

Two other included studies in this systematic review support the results that supplementation promotes the development of a healthier immune system in caesarean-born infants. Both studies found fewer infectious diseases in the caesarean-born intervention group (45, 48). These studies also showed that the differences between the intervention and control groups persisted even after the intervention period. The potential immune modulation of the intervention can be long lasting; meaning that early supplementation can support the immune system to protect against later infectious diseases as found by Peldan et al. (45) after 5–10 years of follow-up. As the follow-up of one year in the study of Puccio et al. (48) was however relatively short, more studies with longer follow up are required to confirm these promising results.

Two of the three studies on immune response to vaccinations after probiotic supplementation found significant effects (46, 47). The immune response to vaccination is a valuable marker reflecting the development of the responsiveness of the immune system to foreign antigens (54, 55). These immunological benefits may be due to an enriched Bifidobacterium population in the gut microbiome. In the literature, an association has been found between reduced abundance of Bifidobacterial species and immune disorders such as pathogenic infections, and allergies (56, 57). Furthermore, an aberrant gut microbiome development has been observed in preterm infants, infants born by CS and after antibiotic exposure in early life, which are all characterized by reduced abundance of Bifidobacterium species (58, 59). Supplementation of a Bifidobacterium probiotic in caesarean-born infants may therefore contribute to a shift in the gut microbiome towards that of vaginally delivered infants, resulting in immunological benefits. However, more studies on the effect of probiotics are needed.

One of the strengths of this review is that, to our knowledge, this is the first review examining the clinical effects of pre-, pro- and synbiotics rather than microbiome differences whose clinical effect is still unclear in caesarean-born infants or infants exposed to antibiotics in the first week of life. One systematic review has recently been published about the effects of probiotics, prebiotics and synbiotics on the microbiome of children born *via* CS (60). However, no clinical outcome measures were reported in this review, which is the ultimate goal for optimizing health in children born following CS or after antibiotic exposure in the first week of life. Furthermore, all full-texts were studied to see if any subgroup analyses of caesarean-born infants were performed, even if this was not explicitly stated in the title or abstract. As a result, only articles that performed analyses on caesarean-born infants were included, and not articles that only analysed the total group of participants, including vaginally born infants.

The main limitation of this review is that nearly all studies were not powered for the clinical outcomes. In most studies, the outcomes for the caesarean-born infants resulted from a subgroup analysis. Moreover, many articles did not adjust for multiple testing, which may have resulted in false positive results. In addition, six of the eight studies scored a high risk of bias, and the included studies were very heterogeneous with regard to the type of supplement studied, the start and duration of the supplementation and the outcome measures. It was therefore not possible to perform a meta-analysis. Furthermore, in the included studies the intervention groups were compared with control groups who received a placebo and, except for one study, not with a “gold standard”: the reference groups of vaginally born and/or breastfed infants that were included in some of the articles. Finally, the follow-up durations of most studies were only one year or less and are therefore too short to investigate the long-term effects.

For future research, several recommendations can be made. Studies need to be adequately powered on clinical outcome measures to investigate the effect of the supplementation. The clinical outcomes of interest, where changes could be expected based on the literature, are: infections, type 1 diabetes, obesity, and atopic diseases such as eczema, allergy, and asthma. These outcome measures need adequate follow-up time. More studies with the same supplement are needed in order to advocate a specific supplement.

## Conclusion

Supplementation of pre-, pro or synbiotics to infants delivered by caesarean section may result in significant improvements in various health outcomes. However, the results were sometimes contradictory or only found in a limited number of studies, and most studies were not adequately powered for the clinical outcome measures. Currently, no studies have been performed examining the effect of supplementation after antibiotic exposure in the first week of life. Due to the variety of study products and the lack of studies, to date no recommendations can be made on how to influence the gut microbiome to improve health outcomes in infants born by caesarean section or with antibiotic exposure in the first week of their life.

## Data Availability

The original contributions presented in the study are included in the article/Supplementary Material, further inquiries can be directed to the corresponding author/s.

## References

[1] Ximenez C Torres J. Development of microbiota in infants and its role in maturation of gut mucosa and immune system. Arch Med Res. (2017) 48(8):666–80. 10.1016/j.arcmed.2017.11.007 29198451

[2] Stewart CJ Ajami NJ O’Brien JL Hutchinson DS Smith DP Wong MC Temporal development of the gut microbiome in early childhood from the TEDDY study. Nature. (2018) 562(7728):583–8. 10.1038/s41586-018-0617-x 30356187 PMC6415775

[3] Sarkar A Yoo JY Valeria Ozorio Dutra S Morgan KH Groer M. The association between early-life gut microbiota and long-term health and diseases. J Clin Med. (2021) 10(3):459. 10.3390/jcm10030459 33504109 PMC7865818

[4] Bokulich NA Chung J Battaglia T Henderson N Jay M Li H Antibiotics, birth mode, and diet shape microbiome maturation during early life. Sci Transl Med. (2016) 8(343):343ra82. 10.1126/scitranslmed.aad7121 27306664 PMC5308924

[5] Wong WS Sabu P Deopujari V Levy S Shah AA Clemency N Prenatal and peripartum exposure to antibiotics and cesarean section delivery are associated with differences in diversity and composition of the infant meconium microbiome. Microorganisms. (2020) 8(2):179. 10.3390/microorganisms8020179 PMC7074690 32012716

[6] Rutayisire E Huang K Liu Y Tao F. The mode of delivery affects the diversity and colonization pattern of the gut microbiota during the first year of infants’ life: a systematic review. BMC Gastroenterol. (2016) 16(1):1–12. 10.1186/s12876-016-0498-0 27475754 PMC4967522

[7] Huurre A Kalliomäki M Rautava S Rinne M Salminen S Isolauri E. Mode of delivery–effects on gut microbiota and humoral immunity. Neonatology. (2008) 93(4):236–40. 10.1159/000111102 18025796

[8] Guo C Zhou Q Li M Zhou L Xu L Zhang Y Breastfeeding restored the gut microbiota in caesarean section infants and lowered the infection risk in early life. BMC Pediatr. (2020) 20(1):532. 10.1186/s12887-020-02433-x 33238955 PMC7690020

[9] Bäckhed F Roswall J Peng Y Feng Q Jia H Kovatcheva-Datchary P Dynamics and stabilization of the human gut microbiome during the first year of life. Cell Host Microbe. (2015) 17(5):690–703. 10.1016/j.chom.2015.04.004 25974306

[10] Jagodzinski A Zielinska E Laczmanski L Hirnle L. The early years of life. Are they influenced by our microbiome? Ginekol Pol. (2019) 90(4):228–32. 10.5603/GP.2019.0041 31059117

[11] Jakobsson HE Abrahamsson TR Jenmalm MC Harris K Quince C Jernberg C Decreased gut microbiota diversity, delayed Bacteroidetes colonisation and reduced Th1 responses in infants delivered by caesarean section. Gut. (2014) 63(4):559–66. 10.1136/gutjnl-2012-303249 23926244

[12] Korpela K de Vos WM. Early life colonization of the human gut: microbes matter everywhere. Curr Opin Microbiol. (2018) 44:70–8. 10.1016/j.mib.2018.06.003 30086431

[13] Munyaka PM Khafipour E Ghia JE. External influence of early childhood establishment of gut microbiota and subsequent health implications. Front Pediatr. (2014) 2:109. 10.3389/fped.2014.00109 25346925 PMC4190989

[14] Hoang DM Levy EI Vandenplas Y. The impact of Caesarean section on the infant gut microbiome. Acta Paediatr. (2021) 110(1):60–7. 10.1111/apa.15501 33405258

[15] Sandall J Tribe RM Avery L Mola G Visser GH Homer CS Short-term and long-term effects of caesarean section on the health of women and children. Lancet. (2018) 392(10155):1349–57. 10.1016/S0140-6736(18)31930-5 30322585

[16] Keag OE Norman JE Stock SJ. Long-term risks and benefits associated with cesarean delivery for mother, baby, and subsequent pregnancies: systematic review and meta-analysis. PLoS Med. (2018) 15(1):e1002494. 10.1371/journal.pmed.1002494 29360829 PMC5779640

[17] Imoto N Kano C Aoyagi Y Morita H Amanuma F Maruyama H Administration of β-lactam antibiotics and delivery method correlate with intestinal abundances of Bifidobacteria and Bacteroides in early infancy, in Japan. Sci Rep. (2021) 11(1):6231. 10.1038/s41598-021-85670-z 33737648 PMC7973812

[18] Korpela K Salonen A Saxen H Nikkonen A Peltola V Jaakkola T Antibiotics in early life associate with specific gut microbiota signatures in a prospective longitudinal infant cohort. Pediatr Res. (2020) 88(3):438–43. 10.1038/s41390-020-0761-5 31954376

[19] Ainonen S Tejesvi MV Mahmud MR Paalanne N Pokka T Li W Antibiotics at birth and later antibiotic courses: effects on gut microbiota. Pediatr Res. (2022) 91(1):154–62. 10.1038/s41390-021-01494-7 33824448 PMC8770115

[20] Rosli R Dali AF Abd Aziz N Abdullah AH Ming LC Manan MM. Drug utilization on neonatal wards: a systematic review of observational studies. Front Pharmacol. (2017) 8:27. 10.3389/fphar.2017.00027 28228724 PMC5297412

[21] van Herk W Stocker M van Rossum AM. Recognising early onset neonatal sepsis: an essential step in appropriate antimicrobial use. J Infect. (2016) 72(Suppl):S77–82. 10.1016/j.jinf.2016.04.026 27222092

[22] Uzan-Yulzari A Turta O Belogolovski A Ziv O Kunz C Perschbacher S Neonatal antibiotic exposure impairs child growth during the first six years of life by perturbing intestinal microbial colonization. Nat Commun. (2021) 12(1):443. 10.1038/s41467-020-20495-4 33500411 PMC7838415

[23] Reyman M van Houten MA Watson RL Chu M Arp K de Waal WJ Effects of early-life antibiotics on the developing infant gut microbiome and resistome: a randomized trial. Nat Commun. (2022) 13(1):893. 10.1038/s41467-022-28525-z 35173154 PMC8850541

[24] Eck A Rutten N Singendonk MMJ Rijkers GT Savelkoul PHM Meijssen CB Neonatal microbiota development and the effect of early life antibiotics are determined by two distinct settler types. PLoS One. (2020) 5;15(2):e0228133. 10.1371/journal.pone.0228133. PMC7001974 32023276

[25] Van Daele E Kamphorst K Vlieger AM Hermes G Milani C Ventura M Effect of antibiotics in the first week of life on faecal microbiota development. Arch Dis Child Fetal Neonatal Ed. (2022):fetalneonatal-2021-322861. 10.1136/archdischild-2021-322861 35534183 PMC9606546

[26] Oosterloo BC van Elburg RM Rutten NB Bunkers CM Crijns CE Meijssen CB Wheezing and infantile colic are associated with neonatal antibiotic treatment. Pediatr Allergy Immunol. (2018) 29(2):151–8. 10.1111/pai.12857 29314334

[27] Alm B Erdes L Mollborg P Pettersson R Norvenius SG Aberg N Neonatal antibiotic treatment is a risk factor for early wheezing. Pediatrics. (2008) 121(4):697–702. 10.1542/peds.2007-1232 18381533

[28] Goksor E Alm B Thengilsdottir H Pettersson R Aberg N Wennergren G. Preschool wheeze—impact of early fish introduction and neonatal antibiotics. Acta Paediatr. (2011) 100(12):1561–6. 10.1111/j.1651-2227.2011.02411.x 21767307

[29] Salvatore S Baldassarre ME Di Mauro A Laforgia N Tafuri S Bianchi FP Neonatal antibiotics and prematurity are associated with an increased risk of functional gastrointestinal disorders in the first year of life. J Pediatr. (2019) 212:44–51. 10.1016/j.jpeds.2019.04.061 31201028

[30] Kamphorst K Oosterloo BC Vlieger AM Rutten NB Bunkers CM Wit EC Antibiotic treatment in the first week of life impacts the growth trajectory in the first year of life in term infants. J Pediatr Gastroenterol Nutr. (2019) 69(1):131–6. 10.1097/MPG.0000000000002360 31058782

[31] Kamphorst K Vlieger AM Oosterloo BC Waarlo S van Elburg RM. Higher risk of allergies at 4–6 years of age after systemic antibiotics in the first week of life. Allergy. (2021) 76(8):2599–02. 10.1111/all.14829 33772817

[32] Kamphorst K Van Daele E Vlieger AM Daams JG Knol J van Elburg RM. Early life antibiotics and childhood gastrointestinal disorders: a systematic review. BMJ Paediatr Open. (2021) 3;5(1):e001028. 10.1136/bmjpo-2021-001028 PMC7931764 33748435

[33] Stromberg Celind F Wennergren G Vasileiadou S Alm B Goksor E. Antibiotics in the first week of life were associated with atopic asthma at 12 years of age. Acta Paediatr. (2018) 107(10):1798–804. 10.1111/apa.14332 29577417 PMC6175332

[34] Goksor E Alm B Pettersson R Mollborg P Erdes L Aberg N Early fish introduction and neonatal antibiotics affect the risk of asthma into school age. Pediatr Allergy Immunol. (2013) 24(4):339–44. 10.1111/pai.12078 23577718 PMC3712479

[35] Roberfroid M. Prebiotics: the concept revisited. J Nutr. (2007) 137(3):830S–7S. 10.1093/jn/137.3.830S 17311983

[36] Moya-Pérez A Luczynski P Renes IB Wang S Borre Y Anthony Ryan C Intervention strategies for cesarean section-induced alterations in the microbiota-gut-brain axis. Nutr Rev. (2017) 75(4):225–40. 10.1093/nutrit/nuw069 28379454 PMC5410982

[37] Rethlefsen ML Kirtley S Waffenschmidt S Ayala AP Moher D Page MJ PRISMA-S: an extension to the PRISMA statement for reporting literature searches in systematic reviews. Syst Rev. (2021) 10(1):1–19. 10.1186/s13643-020-01542-z 33499930 PMC7839230

[38] van Eck NJ Waltman L. Software survey: VOSviewer, a computer program for bibliometric mapping. Scientometrics. (2010) 84(2):523–38. 10.1007/s11192-009-0146-3 20585380 PMC2883932

[39] Wilczynski NL McKibbon KA Haynes RB. Search filter precision can be improved by NOTing out irrelevant content. AMIA Annu Symp Proc. (2011) 2011:1506–13. 22195215 PMC3243169

[40] Ouzzani M Hammady H Fedorowicz Z Elmagarmid A. Rayyan-a web and mobile app for systematic reviews. Syst Rev. (2016) 5(1):210. 10.1186/s13643-016-0384-4 27919275 PMC5139140

[41] Sterne JAC Savovic J Page MJ Elbers RG Blencowe NS Boutron I Rob 2: a revised tool for assessing risk of bias in randomised trials. Br Med J. (2019) 366:l4898. 10.1136/bmj.l4898 31462531

[42] Chua MC Ben-Amor K Lay C Neo AGE Chiang WC Rao R Effect of synbiotic on the gut microbiota of cesarean delivered infants: a randomized, double-blind, multicenter study. J Pediatr Gastroenterol Nutr. (2017) 65(1):102–6. 10.1097/MPG.0000000000001623 28644357

[43] Kallio S Kukkonen AK Savilahti E Kuitunen M. Perinatal probiotic intervention prevented allergic disease in a Caesarean-delivered subgroup at 13-year follow-up. Clin Exp Allergy. (2019) 49(4):506–515. 10.1111/cea.13321 30472801

[44] Kuitunen M Kukkonen K Juntunen-Backman K Korpela R Poussa T Tuure T Probiotics prevent IgE-associated allergy until age 5 years in cesarean-delivered children but not in the total cohort. J Allergy Clin Immunol. (2009) 123(2):335–41. 10.1016/j.jaci.2008.11.019 19135235

[45] Peldan P Kukkonen AK Savilahti E Kuitunen M. Perinatal probiotics decreased eczema up to 10 years of age but at 5–10 years allergic rhino conjunctivitis was increased. Clin Exp Allergy. (2017) 47(7):975–79. 10.1111/cea.12924 28316095

[46] Baglatzi L Gavrili S Stamouli K Zachaki S Favre L Pecquet S Effect of infant formula containing a low dose of the probiotic Bifidobacterium lactis CNCM I-3446 on immune and gut functions in C-section delivered babies: a pilot study. Clin Med Insights Pediatr. (2016) 10:11–9. 10.4137/CMPed.S33096 26997881 PMC4792197

[47] Holscher HD Czerkies LA Cekola P Litov R Benbow M Santema S Bifidobacterium lactis Bb12 enhances intestinal antibody response in formula-fed infants: a randomized, double-blind, controlled trial. JPEN J Parenter Enteral Nutr. (2012) 36(1 Suppl):106S–17S. 10.1177/0148607111430817 22237870

[48] Puccio G Alliet P Cajozzo C Janssens E Corsello G Sprenger N Effects of infant formula with human milk oligosaccharides on growth and morbidity: a randomized multicenter trial. J Pediatr Gastroenterol Nutr. (2017) 64(4):624–31. 10.1097/MPG.0000000000001520 28107288 PMC5378003

[49] Cooper P Bolton KD Velaphi S de Groot N Emady-Azar S Pecquet S Early benefits of a starter formula enriched in prebiotics and probiotics on the gut microbiota of healthy infants born to HIV+ mothers: a randomized double-blind controlled trial. Clin Med Insights Pediatr. (2017) 8;10:119–130. 10.4137/CMPed.S40134 PMC5221488 28096702

[50] Asher M Keil U Anderson H Beasley R Crane J Martinez F International study of asthma and allergies in childhood (ISAAC): rationale and methods. Eur Respir J. (1995) 8(3):483–91. 10.1183/09031936.95.08030483 7789502

[51] Pelucchi C Chatenoud L Turati F Galeone C Moja L Bach J-F Probiotics supplementation during pregnancy or infancy for the prevention of atopic dermatitis: a meta-analysis. Epidemiology. (2012) 23(3):402–14. 10.1097/EDE.0b013e31824d5da2 22441545

[52] Van Der Aa LB Heymans HS Van Aalderen WM Sprikkelman AB. Probiotics and prebiotics in atopic dermatitis: review of the theoretical background and clinical evidence. Pediatr Allergy Immunol. (2010) 21(2p2):e355–67. 10.1111/j.1399-3038.2009.00915.x 19573143

[53] Kelleher MM Cro S Cornelius V Carlsen KCL Skjerven HO Rehbinder EM Skin care interventions in infants for preventing eczema and food allergy. Cochrane Database Syst Rev. (2021) 5:2(2):CD013534. 10.1002/14651858.CD013534.pub2 PMC8094581 33545739

[54] EFSA Panel on Dietetic Products N, Allergies. Guidance on the scientific requirements for health claims related to gut and immune function. EFSA J. (2011) 9(4):1984. 10.2903/j.efsa.2011.1984 PMC13129607 42078786

[55] AlbersRBourdet-SicardRBraunDCalderPCHerzULambertC Monitoring immune modulation by nutrition in the general population: identifying and substantiating effects on human health. Br J Nutr. (2013) 110(S2):S1–S30. 10.1017/S000711451300150523902657

[56] Vatanen T Kostic AD d’Hennezel E Siljander H Franzosa EA Yassour M Variation in microbiome LPS immunogenicity contributes to autoimmunity in humans. Cell. (2016) 165(4):842–53. 10.1016/j.cell.2016.04.007 27133167 PMC4950857

[57] Russell JT Roesch LF Ördberg M Ilonen J Atkinson MA Schatz DA Genetic risk for autoimmunity is associated with distinct changes in the human gut microbiome. Nat Commun. (2019) 10(1):1–12. 10.1038/s41467-019-11460-x 31399563 PMC6689114

[58] Shao Y Forster SC Tsaliki E Vervier K Strang A Simpson N Stunted microbiota and opportunistic pathogen colonization in caesarean-section birth. Nature. (2019) 574(7776):117–21. 10.1038/s41586-019-1560-1 31534227 PMC6894937

[59] Wang S Ryan CA Boyaval P Dempsey EM Ross RP Stanton C. Maternal vertical transmission affecting early-life microbiota development. Trends Microbiol. (2020) 28(1):28–45. 10.1016/j.tim.2019.07.010 31492538

[60] Martin-Pelaez S Cano-Ibanez N Pinto-Gallardo M Amezcua-Prieto C. The impact of probiotics, prebiotics, and synbiotics during pregnancy or lactation on the intestinal microbiota of children born by Cesarean section: a systematic review. Nutrients. (2022) 14;14(2):341. 10.3390/nu14020341 PMC8778982 35057522

