# Supplementary material for: Clinical outcomes following pre-, pro- and synbiotic supplementation after caesarean birth or antibiotic exposure in the first week of life in term born infants: A systematic review of the literature
Source: Front Pediatr. 2022 Oct 10;10:974608. doi: 10.3389/fped.2022.974608 (PMC9589227; doi:10.3389/fped.2022.974608)
Supplement: Supplementary file 1 [file Table1.docx]

| **Supplementary materials** | | |
| --- | --- | --- |
|  | **Ovid MEDLINE(R) ALL <1946 to August 02, 2021> Search date: 3 August 2021** |  |
| **#** | **Searches** | **Results** |
| 1 | exp Cesarean Section/ | 48041 |
| 2 | (C-section* or C?esar?an or Abdominal deliver* or abdominal birth? or (Surg* adj2 deliver*) or (surg* adj2 birth) or Sectio or cesarien or caesarien or postc?esar*).ab,kf,ti. | 69874 |
| 3 | 1 or 2 [cesarean section] | 81595 |
| 4 | exp Anti-Bacterial Agents/ | 758161 |
| 5 | (antibiotic* or anti biotic* or anti bacterial or antibacterial or biocidal or antimicrobial or anti microbial).ab,kf,ti. | 554317 |
| 6 | 4 or 5 [antibiotic treatment] | 1033437 |
| 7 | infant, newborn/ | 627066 |
| 8 | (newborn? or neonat*).ab,kf,ti. | 417342 |
| 9 | (("1" adj1 week?) or (("0" or "1" or "2" or "3" or "4" or "5" or "6" or "7") adj1 day?) or (("12" or "24" or "48" or "72") adj1 hour?)).ab. | 963246 |
| 10 | or/7-9 [first week of life] | 1723901 |
| 11 | 6 and 10 [antibiotic treatment AND first week of life] | 84493 |
| 12 | ((amoxicillin or penicillin G or augmentin or benzylpenicillin or ampicillin) and (gentamicin or amikacin or cefotaxime or ceftazidime)).mp. [1st week antibiotics] | 9134 |
| 13 | or/3,11-12 [children antibiotic treatment c section] | 172617 |
| 14 | prebiotics/ or probiotics/ or synbiotics/ | 21966 |
| 15 | (pre biotic* or pro biotic* or syn biotic* or prebiotic* or probiotic* or synbiotic*).ab,kf,ti. | 35911 |
| 16 | (Arachidonic acid or Bacteroides uniformis or Bifidobacteri* or Bovine-milk derived oligosaccharides or Beta-palmitate or Clostridium butyricum or "E coli" or Escherichia coli or "E faecium L3" or Fructo-oligosaccharides or fructooligosaccharides or Galacto-oligosaccharides or galactooligosaccharides or Inulin-galactooligosaccharide or Oligofructose-inulin or Pectin-derived acidic oligosaccharides or Human milk oligosaccharides or fucosyllactose or fukosyllactose or Lacto-N-neotetraose or Pectin-derived acidic oligosaccharides or Lactobacill* or Lactococcus lactis or Long-chain polyunsaturated fatty acids or Oligofructose-enriched inulin or Polidextrose or Propionibacterium or Propionibacterium freudenreichii or Prebiotic oligosaccharides or Polydextrose or Streptococcus thermophilus).ab,kf,ti. | 421784 |
| 17 | or/14-16 [pre pro synbiotics] | 442980 |
| 18 | exp dietary supplements/ | 85531 |
| 19 | (food? or feed* or diet* or supplement* or nutr*ceutical?).ab,kf,ti. | 1606621 |
| 20 | nutri*.jw. | 245096 |
| 21 | or/18-20 [dietary supplements] | 1734967 |
| 22 | (Aptamil or Biogaia or Probi or Lallemand or Valio or Danone or Nutricia or Nestle or Winclove).ab,in. [dietary supplements manufacturers] | 5610 |
| 23 | 21 or 22 | 1737653 |
| 24 | (Vivomixx or Visbiome or DeSimone Formulation or Danisco-DuPont or Orafti Synergy 1 or oligofructose-enriched inulin or Colinfant or Yakult or Nutrilon or Ecologic Panda).ab,in,kf,ti. [dietary supplements brands] | 894 |
| 25 | (gut or microbi* or gastrointest* or intestin* or flora).mp. [microbiome] | 1983259 |
| 26 | 13 and 17 | 9305 |
| 27 | and/13,23,25 | 4400 |
| 28 | 13 and 24 | 17 |
| 29 | or/26-28 | 12166 |
| 30 | exp animals/ not humans/ | 4868512 |
| 31 | (mouse or mice or rodent? or rat? or pig* or swine? or hog? or cattle or chicken?).ab,kf,ti. | 5199761 |
| 32 | 30 or 31 | 7652474 |
| 33 | 29 not 32 | 7550 |
| 34 | (drug resistance or ((Beta-lactam or Penicillin) adj1 resistan*) or needle biopsy or Burns or Carrier state or Colistin or Emergency service or Endocarditis or Gastroenteritis or Methicillin-resistant staphylococcus aureus or Minocycline or Neoplasms or Peritonitis or Premedication or Prostate or Prosthesis or Surgical wound or Appendicitis or Debridement or Device removal or Drug utilization or Half-life or Hydrocephalus or Middle aged or Preoperative care or Sentinal surveillance or Community-acquired infections or Thienamycins or Naphthyridines or Oxazinesor Oxazolidinones or Tigecycline or Abscess or Drug synergism or Drug tolerance or Sisomicin or Kanamycin or Levofloxacin or Endophthalmitis or Equipment contamination or Microbial sensitivity test*).ab,ti. [VOS cluster 1] | 872358 |
| 35 | (Premature birth or Cholera or Poliovirus vaccine or Pulmonary surfactants or attenuated vaccines or Nitric oxide or Progesterone or Cholera toxin or Anti-hiv agents or "Caco-2 cells" or Cattle or Coculture techniques or animal Disease models or Fetal blood or Germ-free life or Healthy volunteers or ht29 cells).ab,ti. [VOS cluster 2] | 445905 |
| 36 | (Fetal blood or Oryza or Aging or Fetal development or Oxygen consumption or Swine).ab,ti. [VOS cluster 3] | 304932 |
| 37 | (Cardiac surgical procedures or Chewing gum or Gastric bypass or Gastrostomy or Ileostomy or Helicobacter infection? or Helicobactor pylori or Octreotide or Reconstructive surgical procedures or Short bowel syndrome or Anti-ulcer agent? or chewing gum or surgical Anastomosis or Diabetes complication? or Familial duodenal atresia or Gastroschisis or Mesenteric artery or Short bowel syndrome).ab,ti. [VOS cluster 4] | 62632 |
| 38 | (Acne vulgaris or Dental or Drinking water or Fungi or H?ematopoietic stem cell transplantation or Metal nanoparticle? or Precursor cell lymphoblastic leuk?emia-lymphoma or Prosthesis-related infection? or Silver or Typhoid fever or Vegetable? or Waste water or Bone cements or mouthwash* or ophthalmic solutions or root canal or tea or Computer simulation or Ecosystem or Poultry or Poultry diseases or Tissue donor? or X-ray diffraction or Zoonoses).ab,ti. [VOS cluster 5] | 744799 |
| 39 | (molecular cloning or Rabbit?).ab,ti. [VOS cluster 6] | 269631 |
| 40 | (H?emolytic-uremic syndrome or Ambulatory care or Vibrio cholerae).ab,ti. [VOS cluster 7] | 25880 |
| 41 | or/34-40 | 2622024 |
| 42 | 33 not 41 [VOS NOTing out] | 5483 |
|  |  |  |
|  |  |  |
|  | **Ovid Embase Classic+Embase <1947 to 2021 July 30>  Search date: 3 August 2021** |  |
| **#** | **Searches** | **Results** |
| 1 | exp *Cesarean Section/ | 34080 |
| 2 | (C-section* or C?esar?an or Abdominal deliver* or abdominal birth? or (Surg* adj2 deliver*) or (surg* adj2 birth) or Sectio or cesarien or caesarien or postc?esar*).ab,kw,ti. | 107002 |
| 3 | 1 or 2 [cesarean section] | 109703 |
| 4 | exp *antiinfective agent/ | 1631474 |
| 5 | (antibiotic* or anti biotic* or anti bacterial or antibacterial or biocidal or antimicrobial or anti microbial).ab,kw,ti. | 768767 |
| 6 | 4 or 5 [antibiotic treatment] | 2089849 |
| 7 | newborn/ | 642275 |
| 8 | (newborn? or neonat*).ab,kw,ti. | 559799 |
| 9 | (("1" adj1 week?) or (("0" or "1" or "2" or "3" or "4" or "5" or "6" or "7") adj1 day?) or (("12" or "24" or "48" or "72") adj1 hour?)).ab. | 1532622 |
| 10 | or/7-9 [first week of life] | 2326933 |
| 11 | 6 and 10 [antibiotic treatment AND first week of life] | 188981 |
| 12 | ((amoxicillin or penicillin G or augmentin or benzylpenicillin or ampicillin) and (gentamicin or amikacin or cefotaxime or ceftazidime)).mp. [1st week antibiotics] | 75009 |
| 13 | or/3,11-12 [children antibiotic treatment c section] | 364462 |
| 14 | *prebiotic agent/ or *probiotic agent/ or *synbiotic agent/ | 24152 |
| 15 | (pre biotic* or pro biotic* or syn biotic* or prebiotic* or probiotic* or synbiotic*).ab,kw,ti. | 45775 |
| 16 | (Arachidonic acid or Bacteroides uniformis or Bifidobacteri* or Bovine-milk derived oligosaccharides or Beta-palmitate or Clostridium butyricum or "E coli" or Escherichia coli or "E faecium L3" or Fructo-oligosaccharides or fructooligosaccharides or Galacto-oligosaccharides or galactooligosaccharides or Inulin-galactooligosaccharide or Oligofructose-inulin or Pectin-derived acidic oligosaccharides or Human milk oligosaccharides or fucosyllactose or fukosyllactose or Lacto-N-neotetraose or Pectin-derived acidic oligosaccharides or Lactobacill* or Lactococcus lactis or Long-chain polyunsaturated fatty acids or Oligofructose-enriched inulin or Polidextrose or Propionibacterium or Propionibacterium freudenreichii or Prebiotic oligosaccharides or Polydextrose or Streptococcus thermophilus).ab,kw,ti. | 485192 |
| 17 | or/14-16 [pre pro synbiotics] | 510503 |
| 18 | exp dietary supplements/ | 16362 |
| 19 | (food? or feed* or diet* or supplement* or nutr*ceutical?).ab,kw,ti. | 2096249 |
| 20 | nutri*.jx. | 302884 |
| 21 | or/18-20 [dietary supplements] | 2235811 |
| 22 | (Aptamil or Biogaia or Probi or Lallemand or Valio or Danone or Nutricia or Nestle or Winclove).ab,in. [dietary supplements manufacturers] | 11817 |
| 23 | 21 or 22 | 2242040 |
| 24 | (Vivomixx or Visbiome or DeSimone Formulation or Danisco-DuPont or Orafti Synergy 1 or oligofructose-enriched inulin or Colinfant or Yakult or Nutrilon or Ecologic Panda).ab,in,kw,ti. [dietary supplements brands] | 1624 |
| 25 | (gut or microbi* or gastrointest* or intestin* or flora).mp. [microbiome] | 2166306 |
| 26 | 13 and 17 | 21095 |
| 27 | and/13,23,25 | 6238 |
| 28 | 13 and 24 | 40 |
| 29 | or/26-28 | 25508 |
| 30 | (animal/ or animal experiment/ or animal model/ or nonhuman/ or rat/ or mouse/) not human/ | 7159102 |
| 31 | (mouse or mice or rodent? or rat? or pig* or swine? or hog? or cattle or chicken?).ab,kw,ti. | 6979804 |
| 32 | 30 or 31 | 10747161 |
| 33 | 29 not 32 | 12843 |
| 34 | (drug resistance or ((Beta-lactam or Penicillin) adj1 resistan*) or needle biopsy or Burns or Carrier state or Colistin or Emergency service or Endocarditis or Gastroenteritis or Methicillin-resistant staphylococcus aureus or Minocycline or Neoplasms or Peritonitis or Premedication or Prostate or Prosthesis or Surgical wound or Appendicitis or Debridement or Device removal or Drug utilization or Half-life or Hydrocephalus or Middle aged or Preoperative care or Sentinal surveillance or Community-acquired infections or Thienamycins or Naphthyridines or Oxazinesor Oxazolidinones or Tigecycline or Abscess or Drug synergism or Drug tolerance or Sisomicin or Kanamycin or Levofloxacin or Endophthalmitis or Equipment contamination or Microbial sensitivity test*).ab,ti. [VOS cluster 1] | 1217809 |
| 35 | (Premature birth or Cholera or Poliovirus vaccine or Pulmonary surfactants or attenuated vaccines or Nitric oxide or Progesterone or Cholera toxin or Anti-hiv agents or "Caco-2 cells" or Cattle or Coculture techniques or animal Disease models or Fetal blood or Germ-free life or Healthy volunteers or ht29 cells).ab,ti. [VOS cluster 2] | 563387 |
| 36 | (Fetal blood or Oryza or Aging or Fetal development or Oxygen consumption or Swine).ab,ti. [VOS cluster 3] | 390731 |
| 37 | (Cardiac surgical procedures or Chewing gum or Gastric bypass or Gastrostomy or Ileostomy or Helicobacter infection? or Helicobactor pylori or Octreotide or Reconstructive surgical procedures or Short bowel syndrome or Anti-ulcer agent? or chewing gum or surgical Anastomosis or Diabetes complication? or Familial duodenal atresia or Gastroschisis or Mesenteric artery or Short bowel syndrome).ab,ti. [VOS cluster 4] | 99739 |
| 38 | (Acne vulgaris or Dental or Drinking water or Fungi or H?ematopoietic stem cell transplantation or Metal nanoparticle? or Precursor cell lymphoblastic leuk?emia-lymphoma or Prosthesis-related infection? or Silver or Typhoid fever or Vegetable? or Waste water or Bone cements or mouthwash* or ophthalmic solutions or root canal or tea or Computer simulation or Ecosystem or Poultry or Poultry diseases or Tissue donor? or X-ray diffraction or Zoonoses).ab,ti. [VOS cluster 5] | 862062 |
| 39 | (molecular cloning or Rabbit?).ab,ti. [VOS cluster 6] | 362539 |
| 40 | (H?emolytic-uremic syndrome or Ambulatory care or Vibrio cholerae).ab,ti. [VOS cluster 7] | 32757 |
| 41 | or/34-40 | 3390676 |
| 42 | 33 not 41 [VOS NOTing out] | 9149 |
